# Breathing Behaviour Modification of Gallium MIL‐53 Metal–Organic Frameworks Induced by the Bridging Framework Inorganic Anion

**DOI:** 10.1002/chem.202203773

**Published:** 2023-03-13

**Authors:** A. R. Bonity J. Lutton‐Gething, Lynda T. Nangkam, Jens O. W. Johansson, Ioanna Pallikara, Jonathan M. Skelton, George F. S. Whitehead, Inigo Vitorica‐Yrezabal, Martin P. Attfield

**Affiliations:** ^1^ Department of Chemistry The University of Manchester Oxford Road M13 9PL Manchester UK

**Keywords:** breathing, flash heating, metal–organic framework, MIL-53, thermoresponsive behaviour

## Abstract

Controlling aspects of the μ_2_‐X^−^ bridging anion in the metal–organic framework Ga‐MIL‐53 [GaX(bdc)] (X^−^=(OH)^−^ or F^−^, bdc=1, 4‐benzenedicarboxylate) is shown to direct the temperature at which thermally induced breathing transitions of this framework occur. In situ single crystal X‐ray diffraction studies reveal that substituting 20 % of (OH)^−^ in [Ga(OH)(bdc)] (**1**) for F^−^ to produce [Ga(OH)_0.8_F_0.2_(bdc)] (**2**) stabilises the large pore (lp) form relative to the narrow pore (np) form, causing a well‐defined decrease in the onset of the lp to np transition at higher temperatures, and the adsorption/desorption of nitrogen at lower temperatures through np to lp to intermediate (int) pore transitions. These in situ diffraction studies have also yielded a more plausible crystal structure of the int‐[GaX(bdc)] ⋅ H_2_O phases and shown that increasing the heating rate to a flash heating regime can enable the int‐[GaX(bdc)] ⋅ H_2_O to lp‐[GaX(bdc)] transition to occur at a lower temperature than np‐[GaX(bdc)] via an unreported pathway.

## Graphical abstract

**Controlling aspects** of the μ_2_‐X^−^ bridging anion in metal‐organic framework Ga‐MIL‐53 [GaX(bdc)] (X^−^=(OH)^−^ or F^−^, bdc^2−^=terephthalate) can direct the conditions under which the breathing transition of this flexible framework occurs. Substituting 20 % of (OH)^−^ in [Ga(OH)(bdc)] for F^−^ to produce [Ga(OH)_0.8_F_0.2_(bdc)] ⋅ causes changes in the onset of the breathing transition stimulated by temperature or gas adsorption/desorption.

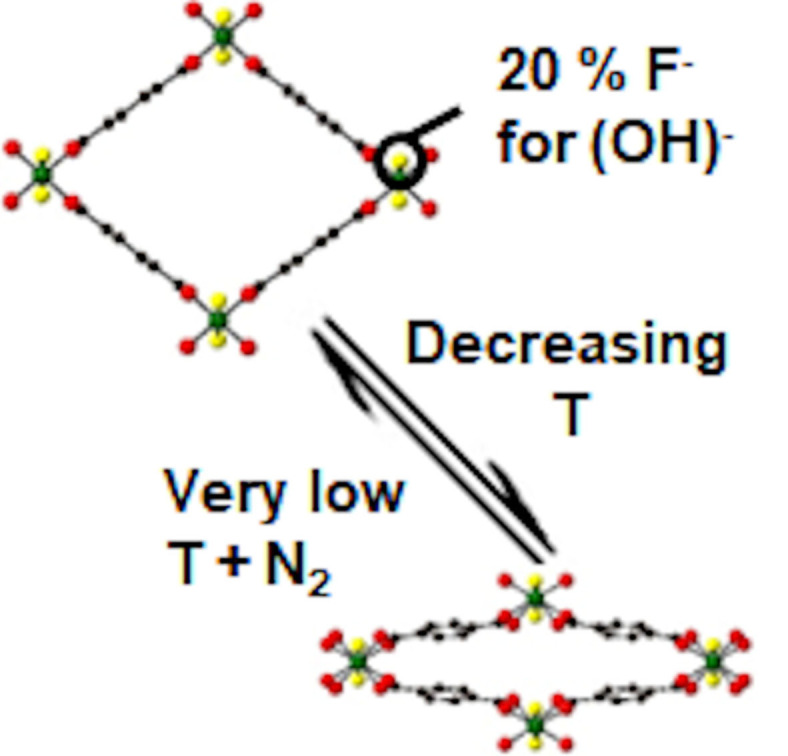

## Introduction

Metal–organic frameworks (MOFs) form the largest family of crystalline porous materials that are receiving a huge amount of interest due to their diverse array of form and function.^[1]^ One subset of MOFs that has received specific interest is breathing or flexible MOFs, whose framework structures change considerably upon the application of external stimuli such as temperature, pressure and host‐guest interactions.^[2]^ This flexible nature furnishes this family of MOF with potential applications that are highly distinctive from rigid porous solids, for example as highly selective flexible adsorbates or stimuli responsive shape selective catalysts.^[3]^

**Figure 1:**
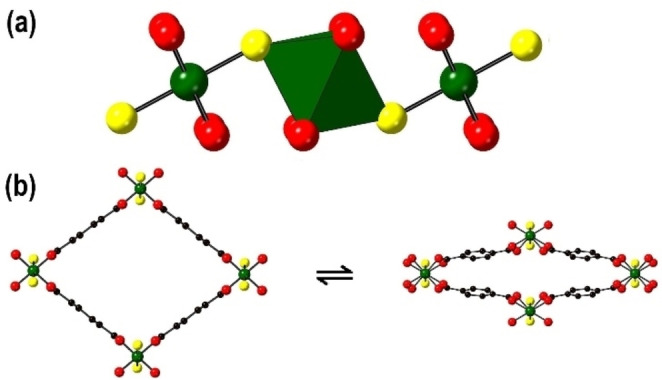
The structures of a constituent chain of *trans‐*corner‐sharing MO_4_X_2_ octahedra (a), and the lp (left) and np (right) phases (b) of flexible MX(bdc). The structures are represented in ball‐and‐stick and polyhedral mode. Atom key: green: M, red: O, yellow: μ_2_‐X^−^ anions=(OH)^−^ or F^−^, black: C, and H atoms are omitted for clarity.

One of the archetypal families of MOF that includes many members that have been shown to exhibit such framework flexibility is the MIL‐53 family^[4]^ and more specifically [MX(bdc)] (where M=Cr,^[5]^ Al,^[6]^ Ga,[^7^, ^8^] V,^[9]^ Fe,^10^ Sc,^[11]^ X^−^=(OH)^−^ or F^−^ and bdc^2−^=1, 4‐benzenedicarboxylate). These materials form from octahedrally‐coordinated trivalent MO_4_X_2_ centres that are connected by μ_2_‐X^−^ anions into 1‐dimensional chains of *trans‐*corner‐sharing MO_4_X_2_ octahedra (see Figure 1a). These chains are bound together by the bdc^2−^ linkers to form a 3‐dimensional framework containing a 1‐dimensional channel system as shown in Figure 1b. MIL‐53 compounds tend to present two main conformations with dramatically different pore volumes that are known as the large pore (lp) and narrow (np) structures as shown in Figure 1b. The lp structure is found at high temperatures, low mechanical pressures or in the presence of large guest molecules or a high pressure of small guest molecules, while the np structure is found under lower temperature or higher mechanical pressure conditions. The inclusion of smaller guest molecules into the pores produce intermediate (int) structures with pore volumes between the lp and np extremes. The size of the pore can be tuned depending on the size and number of the guest molecules.^[4]^

The thermoresponsive breathing behaviour of [MX(bdc)] MIL‐53 MOFs can be directed through changing the trivalent metal, M,[^4^, ^5^, ^6^, ^7^, ^8^, ^9^, ^10^, ^11^] incorporating mixtures of two different metals, M and M’,[^12^, ^13^, ^14^] partially substituting the bdc^2−^ linkers for other dicarboxylate‐based linkers,[^15^, ^16^] or a mixture of these strategies.^[17]^ Another potential route to direct the flexibility of these materials is to change the nature of the μ_2_‐bridging X^−^ anions. A markedly different thermoresponsive behaviour for [Al(X)(bdc)] has been reported. When X^−^=(OH)^−^ the framework is flexible, but when X^−^=F^−^ the framework is rigid.[^6^, ^18^, ^19^] However the difference in thermoresponsive behaviour of the limited number of mixed anion (OH)^−^/F^−^ containing frameworks is less clearly defined.[^20^, ^21^, ^22^] In part, this is due to the fact that the vast majority of [MX(bdc)] samples have been produced in powder form only and not as single crystals. Use of powder samples in diffraction studies results in a broad temperature range over which phase transitions occur. Crystallites in a powder will exhibit a greater range of transition temperatures due to the variability in their size with associated variation in strain and defect content compared to single crystals.[^23^, ^24^] Working with single crystals enables the determination of phase transitions at sharper temperature regimes and higher resolution crystal structures than those obtained from powder samples.

In this work, we use single crystal samples and in situ single crystal X‐ray diffraction to determine detailed structures of guest‐containing and empty MIL‐53 [Ga(OH)(bdc)] (**1**) and [Ga(OH)_0.8_F_0.2_(bdc)] (**2**) frameworks during their thermally induced breathing to demonstrate the significant difference in behaviour of **1** and **2** introduced solely through partial substitution of the μ_2_‐(OH)^−^ anions for μ_2_‐F^−^ anions. We also demonstrate that the heating rate can affect the framework opening transition at lower temperatures via a previously unreported structure transition pathway for this material.

## Results and Discussion

### Crystal structures of 1

All the determined structures are formed of infinite chains of *trans‐*corner‐sharing GaO_4_(OH)_2_ octahedra linked further by the bdc^2−^ ligands to create a 3‐dimensional framework containing 1‐dimensional diamond shaped channels. The octahedrally coordinated Ga centres are bridged by axial‐bound hydroxyl anions, and the oxygen atoms from the bdc^2−^ anions occupy the equatorial positions of the octahedra.

### Crystal structure of int‐1 ⋅ py

**Figure 2:**
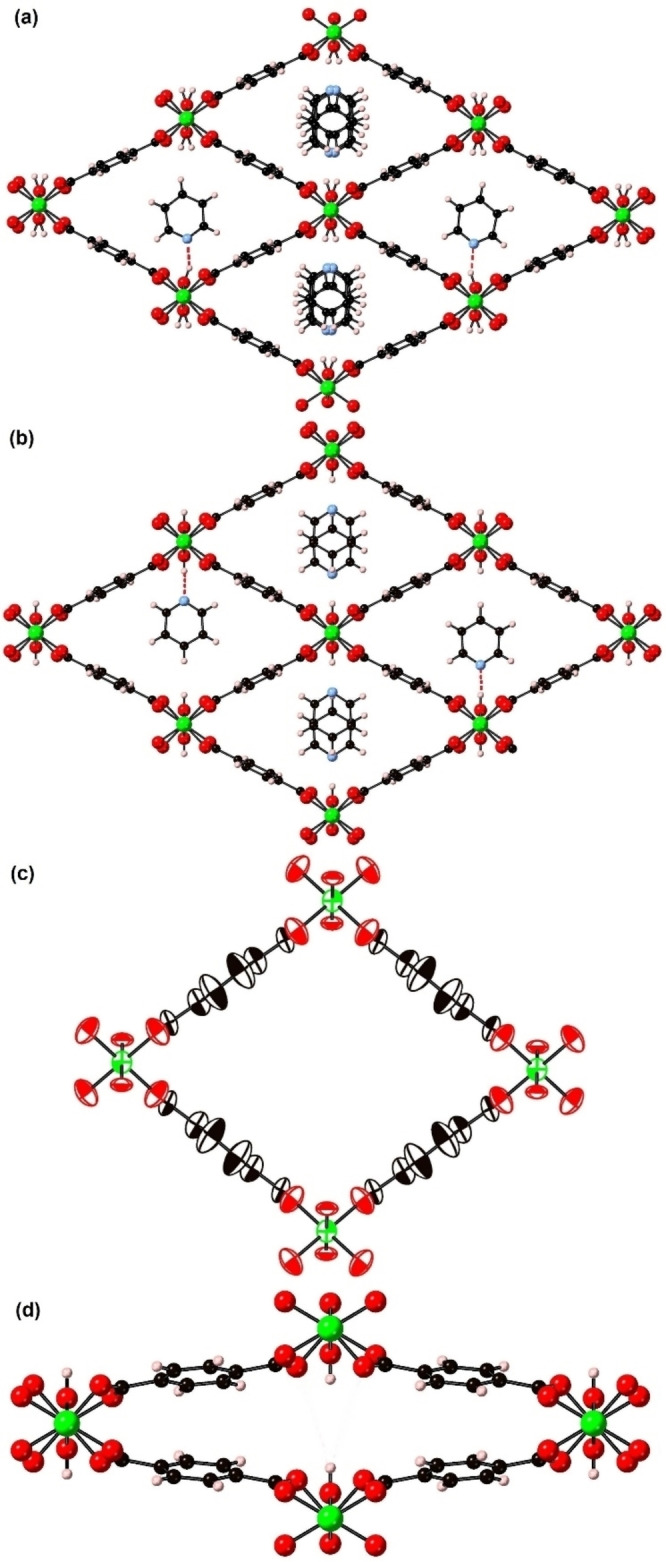
The structure of (a) int‐**1** ⋅ py, (b) int‐**1** ⋅ 0.928(8)py, (c) lp‐**1** and (d) np‐**1**. All the possible orientations of the py molecules are shown in the central two channels of (a) and (b) with different individual orientations shown in the left and right channels. The structures are represented in ball‐and‐stick mode except in (b) where atoms are shown as thermal ellipsoids at 50 % probability. Atom key: green: Ga, red: O, blue: N, black: C, pink: H; and red dashed lines represent N⋅⋅⋅H hydrogen bond (a, b) interactions.

Single crystals of pyridine(py)‐containing int‐**1** ⋅ py were prepared following the reported procedure.^[20]^ The crystal structure of int‐**1** ⋅ py at 150 K is shown in Figure 2a in which the 1‐dimensional channels are fully occupied by py molecules. The plane of the py molecules is perpendicular to the direction of the channel and the plane of the aromatic rings of the bdc^2−^ linkers. The py molecules interact with the framework through hydrogen bond interactions between the N atom of the py and the H atom of the bridging hydroxyl groups (N⋅⋅⋅H 1.82 Å, N⋅⋅⋅O(H) 2.75(1) Å) with weaker π–π and CH–π interactions between adjacent py molecules (separation of py planes 3.37 Å). Both the py guest molecules and corresponding hydrogens of the hydroxyl groups are found to be disordered over two positions. The structure of int‐**1** ⋅ py differs substantially from the reported 298 K int‐[Al(OH)(bdc)] ⋅ py and int‐**2** ⋅ py structures^[20]^ for which the plane of the pore filling py molecules are parallel to the direction of the channel and the plane of the aromatic rings of the bdc^2−^ linkers.

### Thermoresponsive behaviour of int‐1 ⋅ 0.928(8)py

The thermoresponsive behaviour of int‐**1** ⋅ py was monitored using in situ variable temperature single crystal X‐ray diffraction. The initial int‐**1** ⋅ py structure collected at a temperature of 298 K is shown in Figure 2b and shows a 7 % loss of py giving a crystallographically determined chemical formula of int‐**1** ⋅ 0.928(8)py. This structure is similar to int‐**1** ⋅ py (Figure 2a) with the py molecules at each site now occupying one averaged orientation only within the channels. The structure of int‐**1** ⋅ 0.928(8)py is consistent with that of int‐**1** ⋅ 0.85py reported by Vougo‐Zanda et al.^[20]^

Subsequent heating of int‐**1** ⋅ 0.928(8)py to 473 K results in the loss of the py guest molecules and the formation of lp‐**1**. The structure of lp‐**1** at 473 K is shown in Figure 2c in which the framework is seen to be a lp structure with no guest molecules adsorbed into the void space of the material. This is the first structure reported of lp‐**1** and the size and direction of the atomic displacement parameters indicate that the bdc^2−^ linkers are vibrating quite considerably at this temperature. The atomic displacement parameters suggest that the aromatic rings of the bdc^2−^ linkers are oscillating around the central C−C axis of the linker. Such librational bdc^2−^ linker motion has been reported for other bdc^2−^‐containing MOFs, for example MOF‐5.^[25]^ There is a 10.3 % increase in unit cell volume compared to that of int‐**1** ⋅ 0.928(8)py enabled by the loss of py framework interactions.

lp‐**1** was then cooled in 25 K steps to 100 K. The lp‐**1** phase was stable until 325 K, which is the lowest temperature at which the lp‐**1** phase has been isolated as a pure phase. Between 325 K and 300 K a phase transition to the np‐**1** structure was observed. The structure of np‐**1** at 300 K is shown in Figure 2d. The np phase shows additional intra‐framework non‐covalent interactions compared to the lp phase in the form of weak C−H⋅⋅⋅π interactions between the organic linkers along the length of the pore. There is a 40.4 % reduction of the unit cell volume of np‐**1** at 300 K compared to lp‐**1** at 325 K.

The framework remains in the np form on further cooling to 100 K with an accompanying 3.3 % reduction in unit cell volume compared to np‐**1** at 300 K. The crystal was then heated back up to 473 K resulting in a 7.4 % expansion of unit cell volume of the np structure compared to np‐**1** at 100 K.

Full transformation of np‐**1** to lp‐**1** was not observed within the maximum 500 K temperature range accessible with the available instrumentation, suggesting that the non‐covalent interactions within the np form may provide a sufficient energy barrier to prevent the np to lp transition. However, the sample displays clearly the hysteretic behaviour associated with previously reported MIL‐53 frameworks such as [Al(OH)(bdc)]^[26]^ and [Ga(OH)_0.9_F_0.1_(bdc)].^[21]^ The absence of the formation of the lp form at 500 K agrees with the previously reported thermal behaviour of powder **1**, that first indicates the presence of the lp form at ∼473–∼523 K before it becomes the major phase by 613 K.[^7^, ^8^]

The crystals of int‐**1** ⋅ 0.928(8)py studied were generally non‐merohedrally twinned and the number of twin components for this crystal varied for the different phases, with lp‐**1** consisting of two components and np‐**1** consisting of three or four components over the majority of temperatures at which data were collected (see Tables S1, S2). The increased number of twin components on undergoing the lp to np transition reflects the different orientations to which the orthorhombic lp structure can distort on transitioning to the monoclinic np structure.

**Figure 3:**
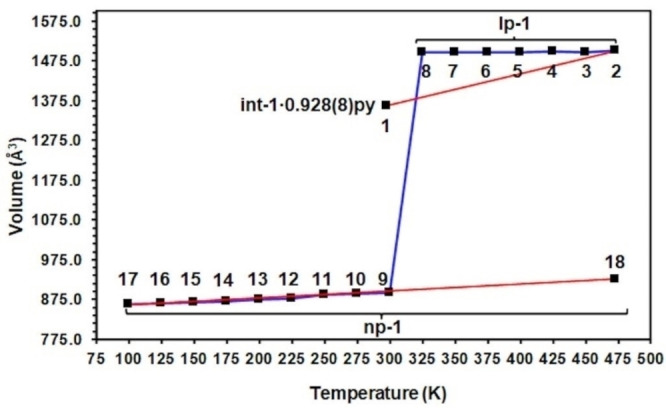
Summary of the thermoresponsive behaviour of int‐**1** ⋅ 0.928(8)py displayed as a plot of unit cell volume against temperature. The order in which the crystal was heated or cooled is indicated by the number associated with each data point, and heating and cooling sections are also represented by the red and blue lines interpolating the data points respectively. The estimated standard deviations for the unit cell volumes lie in the range 0.1–0.5 Å^3^.

A summary of the thermoresponsive behaviour that int‐**1** ⋅ 0.928(8)py undergoes is shown in Figure 3, Figure S4 and full crystallographic information is provided in Tables S1, S2 and the accompanying crystallographic information files (CDDC deposition numbers provided in Table S13). In situ variable temperature single crystal X‐ray diffraction data were collected on another single crystal of int‐**1** ⋅ 0.86(2)py that revealed phase change behaviour and transition temperature points consistent with int‐**1** ⋅ 0.928(8)py.

### Crystal structure of int‐1 ⋅ H_2_O

To further investigate the np to lp transition of the hysteresis cycle of **1**, in situ variable temperature single crystal X‐ray diffraction data were collected for int‐**1** ⋅ H_2_O, where int‐**1** ⋅ H_2_O was considered a good proxy for np‐**1**. Int‐**1** ⋅ H_2_O was prepared as reported in the Experimental Section.

**Figure 4:**
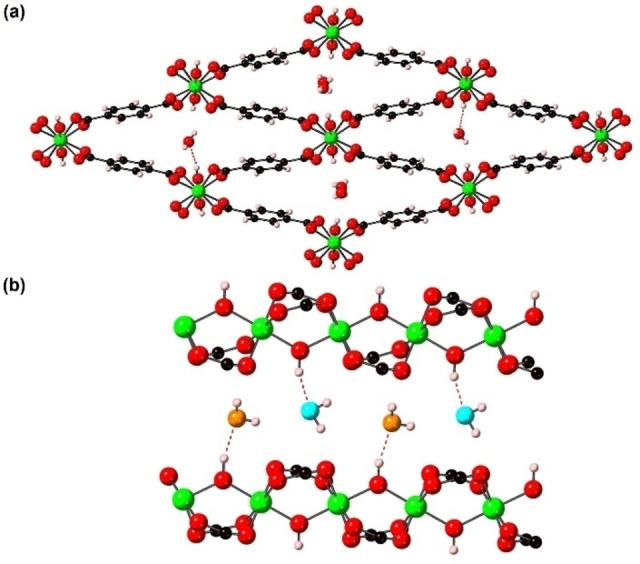
The structure of (a) int‐**1** ⋅ H_2_O and (b) a channel containing the two crystallographically unique water molecules within int‐**1** ⋅ H_2_O viewed perpendicular to the [001] direction. The two crystallographically distinct H_2_O molecules are shown in the central two channels and shown individually in the left and right channels in (a) and as cyan and orange balls in (b). The structure is represented in ball‐and‐stick mode. Atom key: green: Ga, red: O, black: C, pink: H; and red dashed lines represent the shortest H(μ_2_‐OH^−^)…O(H_2_O) hydrogen bond interactions.

The crystal structure of int‐**1** ⋅ H_2_O at 150 K (monoclinic, *P2_1_/c* (see Figure S1), *a*=6.6800(10) Å, *b*=14.8550(7) Å, *c*=19.2691(16) Å, *β*=96.224(3)°, *V*=1900.8(3) Å^3^) is shown in Figure 4a. The framework has an int pore structure with H_2_O molecules adsorbed into the void space of the material. There is a single crystallographically distinct channel, as for int‐**1** ⋅ py, lp‐**1** and np‐**1**, containing two fully occupied distinct H_2_O molecules separated by O⋅⋅⋅O distances of 3.164(4) Å and 3.566(4) Å along the channel as shown in Figure 4b. The H_2_O molecules hydrogen bond to the μ_2_‐bridging (OH)^−^ (O(μ_2_‐OH^−^)⋅⋅⋅O(H_2_O) distances of 2.737(4) Å and 2.760(4) Å for each H_2_O molecule).

This structure is significantly different to that reported previously for this compound and other int‐[M(OH)(bdc)] ⋅ H_2_O analogues.[^7^, ^10^, ^22^, ^27^] To understand why this is, we undertook a detailed comparison of the two systems.

It can be observed in the Ewald sphere projections of the data (Figure S1), systematic absences that agree with the presence of a *c*‐glide perpendicular to the *b* axis (*h*0 *l* absent when *l* is odd). There is also an almost complete set of systematic absences that agree with the presence of an *a*‐glide, perpendicular to *b* axis (*h*0 *l* plane when *h* is odd). However, this is a series of entirely coincidental systematic weaknesses in the diffraction intensity, and it doesn't correspond to a true crystallographic glide plane. We will refer to this as a pseudo‐glide plane.

Transformation of our reported structure to an alternative monoclinic setting in space group *P2_1_/n* (with the *c*‐glide→*n*‐glide and the pseudo *a*‐glide→pseudo *c*‐glide) yields similar unit cell parameters *a*=19.6822(9) Å, *b*=14.8527(8) Å, *c*=6.6754(3) Å, *β*=103.488(5)°, *V*=1897.6(2) Å^3^ to those of the previously reported structures that were determined using space group *P2_1_/c*. This implies that the space group determination for the previously reported structures identified this pseudo *c*‐glide plane as the crystallographic glide plane, missing the true *n*‐glide, as seen in Figure S2. Additionally, the choice of origin of the unit cell differs with the previously reported frameworks containing a metal site fixed on the origin. This results in a model containing three distinct Ga atoms and two different channel types each containing one type of crystallographic H_2_O molecule. Attempts to refine the previously reported structural models[^7^, ^27^] using the data collected in this work, transformed to the corresponding space group setting, result in a surprisingly good fit to the data with an almost comparable R_1_ value to our model (5.4 % versus 5.5 % respectively). However, the refinement is less stable, and the resulting model contains several atoms with negative atomic displacement parameters.

This suggests that the previously reported structures determined using powder X‐ray diffraction data are incorrect. To further confirm this, DFT calculations were used to optimise the transformed int‐**1** ⋅ H_2_O structure reported here (*P2_1_/n*) and the previously reported int‐**1** ⋅ H_2_O structure (*P2_1_/c*).^[27]^ After optimisation, it was found that the framework of the previously reported int‐**1** ⋅ H_2_O (*P2_1_/c*)^[27]^ structure relaxed to a single channel containing framework, as shown in Figure S3a, isostructural to that of our transformed int‐**1** ⋅ H_2_O (*P2_1_/n*) structure. The framework of our transformed int‐**1** ⋅ H_2_O (*P2_1_/n*) structure remained relatively unchanged after optimisation as shown in Figure S3b.

### Thermoresponsive behaviour of int‐1 ⋅ H_2_O

In situ variable temperature single crystal X‐ray diffraction data were collected under a dry N_2_ stream from np‐**1** ⋅ H_2_O at an initial temperature of 298 K. Under these conditions rapid desorption of H_2_O occurred, and the np‐**1** phase was obtained. The crystal was then held at 298 K for 1 hr before heating at a rate of 360 K h^−1^ and collecting data at 350 K, 400 K, 450 K, 475 K and 500 K for which the np‐**1** phase was observed at each temperature. The unit cell volume of np‐**1** at 500 K was 4.6 % larger than at 298 K and closely matched the unit cell volume of the np‐**1** phase at 473 K derived at the end of the variable temperature study of lp‐**1** ⋅ 0.94(2)py (see Tables S3 and S1).

Flash heating was also applied to crystals of int‐**1** ⋅ H_2_O and np‐**1** to determine whether increasing the rate of heating of the crystals would induce the transition to the lp phase. The unit cell of crystals of int‐**1** ⋅ H_2_O held under a stream of dry N_2_ at 100 K were determined (see Table S4) before being directly transferred to a stream of dry N_2_ at 375, 400, 425, 450, 475 or 500 K respectively (see Table S5). The crystal transfered to the stream of dry N_2_ at 500 K transformed directly to lp‐**1** with a 61.8 % increase in equivalent unit cell volume compared to int‐**1** ⋅ H_2_O at 100 K, while the crystals transfered to streams of dry N_2_ at the lower temperatures transformed to np‐**1** only (see Table S5). A similar experiment performed on a crystal of np‐**1** held at a temperature of 353 K under a dry N_2_ stream prior to being directly transferred to a stream of dry N_2_ at 500 K remained in the np‐**1** phase (see Table S6).

### Crystal structures of 2

All the determined structures consist of infinite chains of *trans‐*corner‐sharing GaO_4_(OH)_2_, GaO_4_(OH)F and GaO_4_F_2_ octahedra bound together by the bdc^2−^ linkers to create the 3‐dimensional framework.

### Crystal structure of int‐2 ⋅ 0.84(3)py

**Figure 5:**
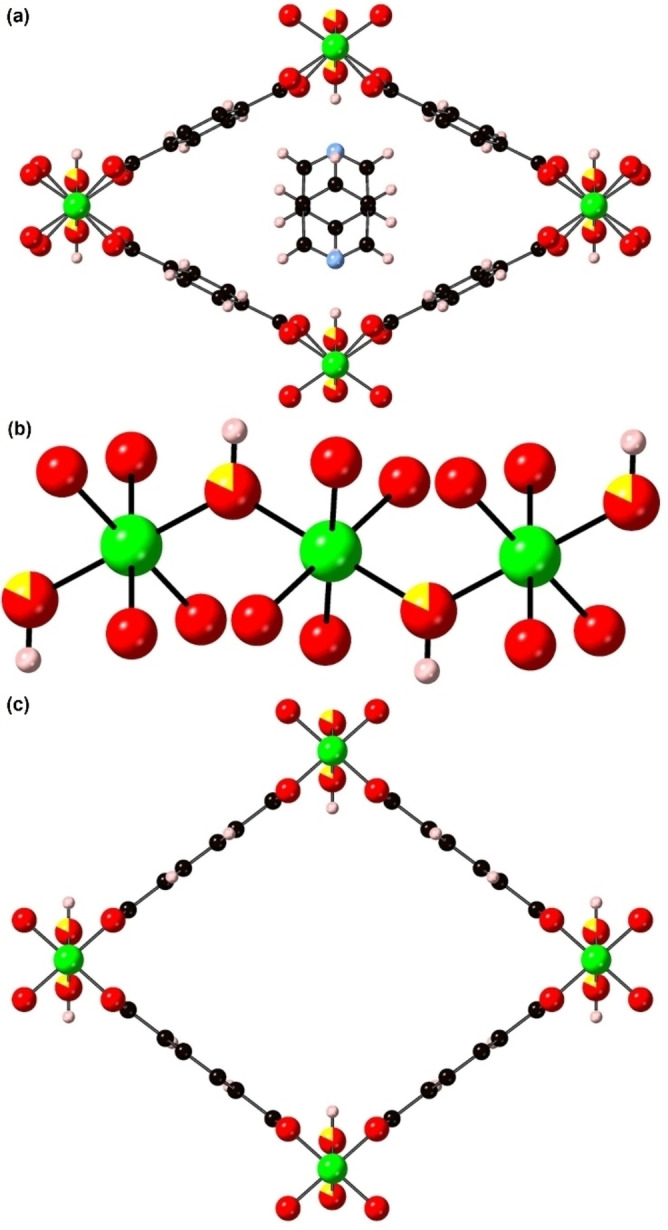
The structure of (a) int‐**2** ⋅ 0.84(3)py, (b) a chain of gallium‐centred octahedra from int‐**2** ⋅ 0.84(3)py and (c) lp‐**2**. Both the possible orientations of the py molecules are shown in (a). The structures are represented in ball‐and‐stick mode. Atom key: green: Ga, yellow: F, red: O, blue: N, black: C, pink: H.

Single crystals of int‐**2** ⋅ 0.84(3)py were prepared following the reported procedure^[20]^ and as detailed in the Experimental Section. The crystal structure of int‐**2** ⋅ 0.84(3)py at 300 K shown in Figure 5a is isostructural to int‐**1** ⋅ 0.94(2)py shown in Figure 2b. The μ_2_‐X^−^ anion site was modelled as a mixed occupancy site containing μ_2_‐(OH)^−^ and μ_2_‐F^−^ anions constrained to have the same atomic coordinates and atomic displacement parameters (see Figure 5b). The occupancies of the μ_2_‐(OH)^−^ and F^−^ atoms were constrained at the values determined from elemental analysis. The framework structure and py location of int‐**2** ⋅ 0.84(3)py is similar to that of int‐**2** ⋅ 0.85py reported previously,^[20]^ however the space group of the latter is *Imma*.

### Thermoresponsive behaviour of int‐2 ⋅ 0.84(3)py

In situ variable temperature single crystal X‐ray diffraction data were collected under a dry N_2_ stream from int‐**2** ⋅ 0.84(3)py at an initial temperature of 300 K. Subsequent heating of int‐**2** ⋅ 0.84(3)py to 475 K resulted in the formation of a major phase (orthorhombic, *Imma*, *a*=17.971(3) Å, *b*=6.691(2) Å, *c*=11.151(3) Å, V=1340.8(6) Å^3^) and a minor phase (orthorhombic, *Imma*, *a*=16.728(18) Å, *b*=6.703(4) Å, *c*=13.08(2) Å, V=1467(3) Å^3^). Continued heating to 500 K resulted in the loss of the py guest molecules and the formation of solely lp‐**2** (previously the minor phase at 475 K) as shown in Figure 5c. The framework is a large pore structure with no guest molecules in the void space. There is an increase in unit cell volume of 9.7 % compared to that of int‐**2** ⋅ 0.84(3)py at 300 K.

lp‐**2** was then cooled in 25 K steps to 100 K. Between 275 and 250 K, the lp to np phase transition was observed, resulting in np‐**2**. This phase transition is observed to occur at a temperature 50 K below that of lp‐**1**. The structure of np‐**2** at 250 K has a 39.3 % reduction of the unit cell volume compared to lp‐**2** at 275 K, similar to that observed for **1**. The lp to np phase transition is accompanied by a significant loss in crystallinity of the sample, with a severe reduction in the quality and resolution of the measurable diffraction data, resulting in a less precise crystal structure of np‐**2** at 250 K than the other structures determined for this crystal. This loss in crystallinity remained for data sets collected at 225 K to 125 K from which only unit cell parameters, and not full structural refinements, were determined.

In contrast to np‐**1**, the crystal of np‐**2** undergoes a np to lp phase transition as it is cooled further between 125 and 100 K, resulting in the formation of lp‐**2** ⋅ ∼4 N_2_, which is isostructural with the higher temperature lp‐**2** structures. This phase transition is accompanied by a significant improvement in the crystallinity of the sample, allowing a high resolution dataset to be collected. The framework structure of lp‐**2** ⋅ ∼4 N_2_ at 100 K is similar to the framework shown in Figure 5c with the void space now being occupied by adsorbed N_2_ molecules. The N_2_ molecules were not sufficiently ordered within the pore space of the framework to be determined crystallographically. However, solvent mask calculations^[28]^ indicated an electron density equivalent to 222 electrons was present in the void volume of the unit cell that approximates to ∼16 N_2_ molecules, giving the chemical formula **2** ⋅ ∼4 N_2_. The number of N_2_ molecules in the formula appears over estimated in comparison to the chemical formulae **1** ⋅ ∼3.1–3.5 N_2_ obtained from 77 K nitrogen adsorption measurements.[^7^, ^8^] No nitrogen adsorption data has been reported for **2**, so those reported for **1** are used in this comparison due to the expected similarity in behaviour of **1** and **2** in terms of the amount of N_2_ adsorbed. This transition during the drop in temperature of the crystal is the result of greater adsorption of N_2_ gas molecules from the N_2_ gas stream within the pores of the framework as temperature drops. Similar lp to np to lp transitions have been predicted and experimentally supported using gas adsorption isotherms for the adsorption of Xe by [Al(OH)(bdc)],^[29]^ but this provides the first structural data for such a set of transitions.

The crystal was then heated from 100 K in 25 K steps and was found to retain the lp form with N_2_ gas adsorbed until 150 K, indicative of the hysteretic desorption behaviour associated with these flexible MIL‐53 materials.^[30]^ From 150 to 175 K, the lp form was found to partially collapse to an intermediate int‐**2** phase at 175 K. This is a partial transformation of the framework as there is a 25.2 % reduction of the unit cell volume, rather than the ∼40 % decrease typically observed for the lp to np phase transition.

This transition during the rise in temperature of the crystal is probably instigated by the desorption of N_2_ gas molecules from the pores of the framework. This phase transition from the lp phase is again accompanied by a sharp loss in crystallinity of the crystal and a severe reduction in the quality and resolution of diffraction data collected, resulting in only unit cell parameters being determined at 175 K. Above 175 K the crystallinity of the crystal had deteriorated to the extent that no diffraction data could be collected at higher temperatures.

The crystals of int‐**2** ⋅ 0.84(3)py studied were generally non‐merohedrally twinned and the number and fractional amount of the twin components varied for the different phases of this crystal (see Tables S7, S8). Of note is that between 500 and 275 K the crystal consisted of one dominant lp‐**2** component, that presumably formed a greater number of less ordered and misaligned components on undergoing the lp to np transition. This reflects the different orientations to which the orthorhombic lp structure can distort on transitioning to the monoclinic np structure. However, on further cooling to 100 K and undergoing the monoclinic np to orthorhombic lp transition, these components realigned to a significant extent resulting in a strongly diffracting crystal again. The crystal of lp‐**2** ⋅ ∼4 N_2_ between 100 and 150 K consisted of three major components compared to the one major component in lp‐**2** between 500 and 275 K, indicating imperfect realignment of components, or possible cracking of the crystal, upon re‐transitioning to the lp phase. This demonstrates the instability of the **2** MIL‐53 crystals to multiple breathing transitions that is unsurprising when the number of domains in the crystal and the possible ways that they can transform during these breathing transitions are considered. This will result in their low likelihood to remain intact and aligned during many phase transitions.

**Figure 6:**
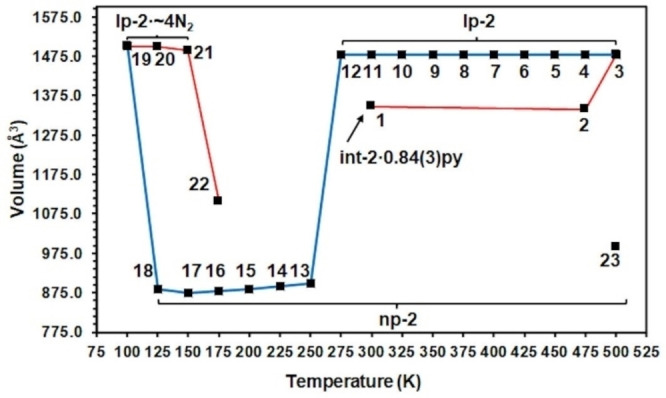
Summary of the thermoresponsive behaviour of int‐**2** ⋅ 0.84(3)py displayed as a plot of unit cell volume against temperature. The order in which the crystal was heated or cooled is indicated by the number associated with each data point, and heating and cooling sections are also represented by the red and blue lines interpolating the data points respectively. The estimated standard deviations for the unit cell volumes lie in the range 0.1–4 Å^3^. Point 23 is taken from the study of the thermoresponsive behaviour of int‐**2** ⋅ H_2_O.

A summary of the thermoresponsive behaviour that int‐**2** ⋅ 0.84(3)py undergoes is shown in Figure 6, Figure S4 and full crystallographic information is provided in Table S7, S8 and CCDC deposition numbers for the accompanying crystallographic information files are given in Table S13. In situ variable temperature single crystal X‐ray diffraction data were collected on another single crystal of int‐**2** ⋅ 0.80(9)py that revealed similar phase change behaviour and transition temperature points within 25 K of the first crystal.

The slight variability in the transition temperature points between individual crystals is likely to be linked to a difference in the amount of μ_2_‐F^−^ anions present in the framework. This is supported by the EDX analysis over several crystals which revealed that the Ga : F ratio in the sample varied between 0.13–0.23. However, other structural features such as the number of crystal domains and their relative orientation could also contribute to the variation in thermoresponsive phase change behaviour observed. The reported behaviour of the lp‐**2** to np‐**2** transition is supported by the reported behaviour of powder [Ga(OH)_0.9_F_0.1_(bdc)] that remains predominantly in the lp‐form on cooling from 573 K to 303 K.^[21]^

### Crystal structure of int‐2 ⋅ H_2_O

To further investigate the np to lp transition of the hysteresis cycle of **2**, in situ variable temperature single crystal X‐ray diffraction data were collected for int‐**2** ⋅ H_2_O, where int‐**2** ⋅ H_2_O was again considered a good proxy for np‐**2**. Int‐**2** ⋅ H_2_O was prepared as reported in the Experimental Section.

**Figure 7:**
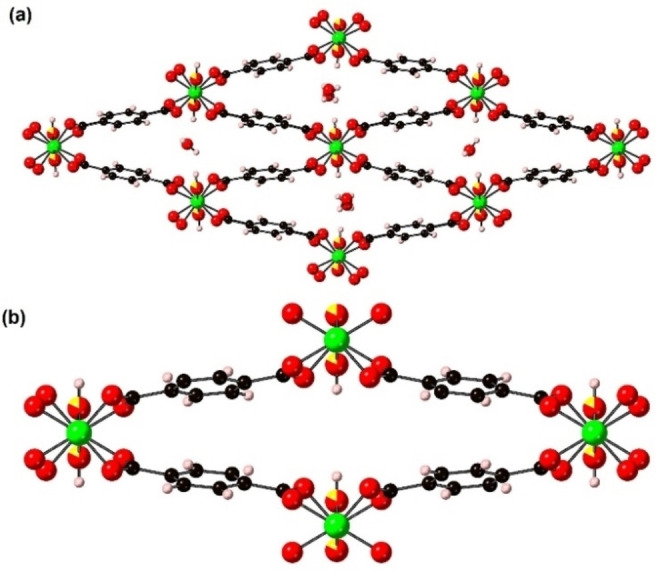
The structure of (a) int‐**2** ⋅ H_2_O and (b) np‐**2**. The two crystallographically distinct H_2_O molecules in (a) are shown in the central two channels and shown individually in the left and right channels for clarity. The structures are represented in ball‐and‐stick. Atom key: green: Ga, yellow: F, red: O, black: C, pink: H.

The crystal structure of int‐**2** ⋅ H_2_O at 100 K (monoclinic, *P2_1_/c*, *a*=6.6662(3) Å, *b*=14.7953(10) Å, *c*=19.2119(8) Å, *β*=96.366(4)°, *V*=1883.16(17) Å^3^) is shown in Figure 7a and is isostructural to int‐**1** ⋅ H_2_O. This is the first report of a int‐[Ga(OH)_0.8–0.9_F_0.1–0.2_(bdc)] ⋅ H_2_O structure with this unit cell. The previous crystal structure reported has a smaller unit cell that may be due to the systemic weakness of any Bragg peaks that would result in a doubling of the *b* axis for this unit cell.^[21]^

### Thermoresponsive behaviour of int‐2 ⋅ H_2_O

In situ variable temperature single crystal X‐ray diffraction data were collected under a dry N_2_ stream from int‐**2** ⋅ H_2_O at an initial temperature of 350 K. Under these conditions rapid desorption of H_2_O had occurred, and the np‐**2** phase was obtained. The crystal was then heated to 500 K in 25 K steps for which the np‐**2** ⋅ phase was observed at each temperature (see Table S9). The structure of np‐**2** at 500 K is shown in Figure 7b and the unit cell volume is 4 % larger than at 350 K (see Table S9). Full transformation of np‐**2** to lp‐**2** was not observed over the temperature range accessible with the available instrumentation. It is also interesting to note that the crystallinity of np‐**2** derived from int‐**2** ⋅ H_2_O is much greater than that of np‐**2** derived from the in situ study of int‐**2** ⋅ 0.84(3)py. This is presumably due to the different routes of formation of np‐**2**, which likely occurs via lp‐**2** to int‐**2** ⋅ H_2_O to np‐**2** host‐guest induced transitions for the former and a direct lp‐**2** to np‐**2** thermally induced transition for the latter.

The thermal stability of both np‐**2** and lp‐**2** phases through the 500–250 K temperature range demonstrate the hysteretic behaviour of **2** associated with MIL‐53 frameworks. The absence of the formation of the lp form at 500 K agrees with that reported for the thermal behaviour of powder [Ga(OH)_0.9_F_0.1_(bdc)] that first indicates the presence of the lp form at ∼423 K before it becomes the major phase by 523 K.^[21]^

Flash heating was also applied to crystals of int‐**2** ⋅ H_2_O initially held at 150 or 100 K (see Table S10) and np‐**2** initially held at 350 K (see Table S12). int‐**2** ⋅ H_2_O transformed directly to lp‐**2** at 500 K only, with a 59.4 % increase in unit cell volume (see Table S11). np‐**2** did not undergo a phase transition to lp‐**2** (see Table S12).

### Comparison of the thermoresponsive behaviours of 1 and 2

The results demonstrate that inclusion of μ_2_‐F^−^ anions into the framework affects the thermoresponsive behaviour of [Ga(X)bdc]. A ∼20 % inclusion of F^−^ into the framework of Ga‐MIL‐53 induces a ∼50–75 K decrease in the onset of the lp to np phase transition at higher temperatures (∼250–325 K) and instigates np to lp to int phase transitions upon cooling and heating with associated probable adsorption/desorption of N_2_ at lower temperatures (∼100–175 K) (see Figure S4). These changes in behaviour relate directly to the reduction in stability of the np‐**2** phase relative to the lp‐**2** phase when compared to the np‐**1** and lp‐**1** phases upon the introduction of μ_2_‐F^−^ anions in the framework. The reduction in stability of the np‐**2** phase relative to the lp‐**2** phase will derive from the additional distortions and strain produced in, and by, the chains of gallium‐centred octahedra, when GaO_4_(OH)_2_ are replaced by GaO_4_(OH)F or GaO_4_F_2_ centres. Such distortions will be introduced through the electronic differences in the F^−^ and (OH)^−^ ions and the resulting effect on the bonding within the framework. The greater difference in strain induced by F^−^ ion inclusion within the chains of gallium‐centred octahedra is demonstrated by the loss of crystallinity for **2** during the lp to np transition as compared to **1**.

The results also demonstrate that both int‐**1** ⋅ H_2_O and int‐**2** ⋅ H_2_O transform to their lp phases upon flash heating thus completing the hysteresis cycle of **1** and **2**. However, np‐**1** and np‐**2** do not transform to their lp phases upon flash heating which indicates a strong influence of the presence of guest molecules in this process. The int to lp transition appears to occur without passing through the np phase within the time resolution of this experiment, which contrasts with the behaviour of powder samples of these materials that convert under a slower rate of heating to the np phase before transforming to the lp phase.[^7^, ^8^, ^21^] Flash heating thus provides a new route to the lp phase under these conditions. The results would suggest the route for this transformation to occur is by any of the pathways:

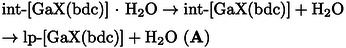




or

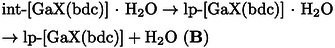




or

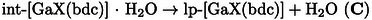




However, more temporally resolved diffraction data would need to be collected along with information from other techniques to determine which of the sequences is most representative.

The ability of int‐[GaX(bdc)] ⋅ H_2_O, and not np‐[GaX(bdc)], to transform into the lp phase upon flash heating is presumably connected to the framework of int‐[GaX(bdc)] ⋅ H_2_O being slightly more open than np‐[GaX(bdc)], reducing the activation energy required for the transformation to the lp structure. This is similar to the transitions seen for int‐[GaX(bdc)] ⋅ *x*py, which lowers the temperature required to access the lp phase to 473–475 K. This implies that int‐[GaX(bdc)] ⋅ H_2_O has a higher barrier to transition to lp than int‐[GaX(bdc)] ⋅ py.

The fact that the transformation occurs at 500 K, and not below, suggests that only at 500 K is there enough thermal energy for the rapid structure transition to lp via one of the above pathways **A**–**C**. Pathways **B** or **C** may be the more likely pathways as below 500 K water is readily lost to form the intermediate in pathway **A** (int‐[GaX(bdc)]) before the structure transition to the favoured np structure. This suggests the presence of guest H_2_O, to provide alternative energetic transition pathways, and high temperatures, to provide sufficient thermal energy, are both necessary for the int to lp transition to proceed under flash heating conditions.

Similar to the int‐[GaX(bdc)] ⋅ py transition, the flash heating transformation of both int‐**1** ⋅ H_2_O and int‐**2** ⋅ H_2_O have no apparent temperature differential for the transition being introduced by the incorporation of μ_2_‐F^−^ anions in the framework. This suggests the activation energy for the high temperature opening process is similar for both **1** and **2** and is not dictated by the nature of the μ_2_‐X^−^ anion but is instead related to the relative strain and intermolecular interactions of the bdc^2−^ linkers and interactions involving the guest molecules.

## Conclusions

The use of in situ single crystal X‐ray diffraction has revealed the changes in the thermally induced breathing transitions of Ga‐MIL‐53 MOF [GaX(bdc)] caused by changing the μ_2_‐X^−^ anion from (OH)^−^ to F^−^. A 20 % substitution of (OH)^−^ for F^−^ stabilises the lp form relative to the np form causing a ∼50–75 K decrease in the onset of the lp to np phase transition at higher temperatures during the cooling part of the hysteresis cycle. It also instigates np to lp to int phase transitions upon cooling and heating with associated adsorption/desorption of N_2_ at lower temperatures (∼100–175 K). Controlling the degree of substitution and nature of the μ_2_‐X^−^ anion in this MIL‐53 family and possibly other families of MOF can provide a direct method to tune the onset of framework flexing behaviour, with potential applications in molecule sensing, separation, and storage. The work has also enabled the determination of the true crystal structure of the int‐[MX(bdc)] ⋅ H_2_O phases for the MIL‐53 family and shown that increasing the heating rate to a flash heating regime can enable the framework opening transition for int‐[GaX(bdc)] ⋅ H_2_O to occur at a lower temperature than np‐[GaX(bdc)] via a previously unreported structure transition pathway. Application of rapid heating rates or flash heating to other flexible MOFs and extended solids may yield an effective strategy to access the higher energy forms of these compounds at lower temperatures.

## Experimental Section

**General**: Ga(NO_3_)_3_ ⋅ xH_2_O (Alfa Asear, 99 %), HF (Honeywell, 48 % in H_2_O), dimethylformamide (DMF, Fisher Scientific, 99.5 %), pyridine (py, C_5_H_5_N, Alfa Asear. 99 +%) and terephthalic acid (H_2_bdc, Sigma Aldrich, 98 %) were used as received without any further purification. Laboratory tap water was purified using a Milli‐Q system (18 MΩ cm resistivity at 25 °C) before use.

### Syntheses

**Synthesis of int‐1 ⋅** ***x*** **py**: int‐**1** ⋅ py was synthesised via int‐**1** ⋅ 0.74H_2_bdc according to the methods reported by Vougo‐Zanda et al.^[20]^ 0.33 g (2 mmol) H_2_bdc, 0.27 g (1 mmol) Ga(NO_3_)_3_ ⋅ xH_2_O (assuming x=zero) and 10 mL deionised water were placed in a 23 mL Teflon lined autoclave and heated in an oven at 493 K for 72 hr. The resulting white solid was collected by suction filtration and washed with 20 mL of heated DMF followed by 10 mL of deionised water. The white solid consisted of both long needles of crystalline H_2_bdc and block crystals of the int‐**1** ⋅ 0.74H_2_bdc.

Approximately 0.2 g of int‐**1** ⋅ 0.74H_2_bdc was placed in a Teflon lined 23 mL autoclave followed by 10 mL of pyridine and placed in a pre‐heated oven at 433 K for 5 days. Once cooled to room temperature block crystals of int‐**1** ⋅ py were collected by suction filtration and washed with two 10 mL portions of pyridine. Crystals were kept in py to produce int‐**1** ⋅ py. Crystals of int‐**1** ⋅ py left in air gave int‐**1** ⋅ 0.928(8)py.

**Synthesis of int‐1 ⋅ H_2_O**: Approximately 0.2 g of int‐**1** ⋅ py was placed in a crucible and heated in a tube furnace under N_2_ gas at a rate of 0.1° min^−1^ to 463 K before being held at this temperature for 10 hr. The sample was then cooled at a rate of 0.1° min^−1^ to room temperature and exposed to atmospheric air resulting in adsorption of atmospheric water.

**Synthesis of int‐2 ⋅ 0.88(3)py and int‐2 ⋅ H_2_O**: int‐**2** ⋅ 0.88(3)py and int‐**2** ⋅ H_2_O were synthesised following the same method as for int‐**1** ⋅ 0.74H_2_bdc, int‐**1** ⋅ py and int‐**1** ⋅ H_2_O except for the addition of 1.5 mmol (48 mg, 48 %) HF to the initial reagent mixture to prepare int‐**2** ⋅ 0.74H_2_bdc as described in the method reported by Vougo‐Zanda et al.^[20]^ A FEI Quanta 200 ESEM microscope operated under low vacuum (0.3 Torr) with an accelerating voltage of 15 kV (approximate beam diameter of 1 μm) with an EDX Genesis energy dispersive X‐ray spectrometry system was used to determine the Ga : F ratio of 1 : 0.2 that closely matched the ratio reported by Vougo‐Zanda et al.^[20]^

**Single crystal X‐ray diffraction**: Single crystals of int‐**1** ⋅ py, int‐**1** ⋅ 0.928(8)py, int‐**2** ⋅ 0.84(3)py, int‐**1** ⋅ H_2_O, or int‐**2** ⋅ H_2_O were selected and directly mounted on a MiTeGen Polymer loop without any adhesive agent. Crystals were mounted on a Rigaku Oxford FR‐X diffractometer fitted with a rotating anode X‐ray dual wavelength source. A Cyrostream 800 Plus was used to vary the temperature of the crystals at a rate of 360 K hr^−1^ and keep them under a flow of dry N_2_(g) during data collection. Diffraction data were collected using Cu or Mo Κα radiation and the CrysAlisPro suite of programs, and data were reduced using CrysAlisPro software (Rigaku, v40.69a). Absorption corrections were performed using empirical methods based upon symmetry‐equivalent reflections combined with measurements at different azimuthal angles as implemented by SCALE3 ABSPACK. Structure solution and refinement was preformed against all *F* ^2^ values using ShelxT^[31]^ and ShelxL^[32]^ respectively, through the Olex2(v1.5)^[33]^ interface.

Twinning or multiple domains were observed in the diffraction patterns for the majority of the crystals. The different approaches used to determine the crystal structures from these crystals are as follows:

(i) The relationship between the twin domains was determined using CrysAlisPro (Rigaku, v40.69a), software by inspection of the reflections using an Ewald sphere projection, followed by the assignment of reflections to multiple domains. Two hkl files were produced upon data reduction; one hklf4 format containing non‐overlapped reflections from a single component and one hklf5 format containing the non‐overlapping and overlapping reflections for both components. The hklf4 file was then used for subsequent structural solution and the hklf5 file for refinement.

(ii) Only the major component was indexed and the data reduced to produce a single hklf4 format hkl file, temporarily ignoring reflection overlap from other crystal domains. Following the structure solution, the TwinRotMat program (available in Platon),^[34]^ was used to determine a twin law to account for domain overlap, generating an hklf5 format hkl file. The model was then refined using the new file accounting for the twin domain overlap. This method was used where the major component significantly dominated the diffraction pattern but where the presence of minor components was clear, resulting in a poor integration when using method (i).

(iii) Data reduction was performed on the major component and structure determination was determined from this domain without any further allowance for the other domains in the crystal. This method was used when there was no obvious minor components to index, despite some contributions to the diffraction intensity from additional components.

All non‐hydrogen atoms were refined using anisotropic displacement parameters or isotropic displacement parameters where this was not possible. Hydrogen atoms were either located from difference Fourier maps or placed in calculated positions and refined using idealized geometries (a riding model), assigned fixed isotropic displacement parameters and occupancies. The C_5_H_5_N atoms of the pyr molecules in the relevant crystal structures were linked to a free variable and the occupancy of the pyr molecule allowed to refine freely. Various geometric and atomic displacement parameter restraints on the framework and non‐framework organic components of the crystal structures were applied as required to obtain the final crystal structures as detailed in the crystallographic information files for each crystal structure. For structures where solvent is particularly disordered the Squeeze^[28]^ procedure was applied to estimate the electrons within the void space.

Deposition Numbers 2223704‐ 2223707, 2223710, 2223711, 2223716, 2223721–2223733, and 2223735–2223753 (see Table S13) contain the supplementary crystallographic data for this paper. These data are provided free of charge by the joint Cambridge Crystallographic Data Centre and Fachinformationszentrum Karlsruhe Access Structures service.

**Computational simulations**: Pseudopotential plane wave density functional theory calculations were performed to compare the stability of the structure of int‐**1** ⋅ H_2_O reported here and that reported previously^[27]^ using the Vienna *Ab Initio* Simulation Package (VASP) code.[^35^, ^36^, ^37^] The PBEsol^[38]^ generalised gradient approximation functional (GGA) with a DFT‐D3^[39]^ dispersion correction were used to describe electron exchange and correlation. Ion cores were modelled using pseudopotentials generated using the projector augmented wave (PAW) method[^40^, ^41^] with Ga 4s/3d/4p, O 2s/2p, C 2s/2p and H 1s electrons treated as valence states. The starting models for the optimisations were the structure of int‐**1** ⋅ H_2_O reported previously^[27]^ (CCDC 1007163) and the int‐**1** ⋅ H_2_O reported in this work that had been transformed to the same unit cell setting (monoclinic, *P2_1_/n*, *a*=19.6822(9) Å, *b*=14.8527(8) Å, *c*=6.6754(3) Å *β*=103.488(5)°, *V*=1897.6(2) Å^3^). For both structures, the electronic Brillouin zones were sampled using a Γ‐centred Monkhorst‐Pack ***k***‐point mesh^[42]^ with 1 x 1 x 2 subdivisions. Valence wavefunctions were described using a plane wave basis with kinetic energy cut off values of 800 eV. Full geometry relaxations, including all unit cell lengths, the non‐90° angle, unit cell volume and atomic positions were performed for both structures. A tolerance of 10^−2^ eV Å^−1^ on the ionic forces was applied during geometry optimisations.

## Conflict of interest

The authors declare no conflict of interest.

1

## Supporting information

As a service to our authors and readers, this journal provides supporting information supplied by the authors. Such materials are peer reviewed and may be re‐organized for online delivery, but are not copy‐edited or typeset. Technical support issues arising from supporting information (other than missing files) should be addressed to the authors.

Supporting Information

## Data Availability

The data that support the findings of this study are available in the supplementary material of this article.
