# Supplementary material for: Breathing Behaviour Modification of Gallium MIL‐53 Metal–Organic Frameworks Induced by the Bridging Framework Inorganic Anion
Source: Chemistry. 2023 Mar 13;29(21):e202203773. doi: 10.1002/chem.202203773 (PMC10962686; doi:10.1002/chem.202203773)
Supplement: Supplementary file 1 — Supporting Information [file CHEM-29-0-s001.pdf]

Chemistry–A European Journal

Supporting Information

Breathing Behaviour Modification of Gallium MIL-53 Metal–Organic Frameworks Induced by the Bridging Framework Inorganic Anion

A. R. Bonity J. Lutton-Gething, Lynda T. Nangkam, Jens O. W. Johansson, Ioanna Pallikara, Jonathan M. Skelton, George F. S. Whitehead, Inigo Vitorica-Yrezabal, and Martin P. Attfield*

Table S1: Summary of unit cell parameters and observed domains for the variable temperature data collection for int-**1**·0.928(8)py over the temperature range 100 - 473 K.

**Sequence Number (corresponding to Figure 3)**	**Temperature/ K**	**Space group**	***a*/ Å**	***b*/ Å**	***c*/ Å**	***β*/ °**	**Volume/ Å³**	**Number of Domains**
1	298	*I2/a*	18.1118(7)	11.1397(6)	6.7515(3)	91.792(3)	1361.52(11)	2
2	473	*Imma*	16.7753(17)	6.7401(7)	13.2673(18)	90	1500.1(3)	4
3	450	*Imma*	16.7462(6)	6.7405(2)	13.2584(5)	90	1496.58(9)	2
4	425	*Imma*	16.7512(7)	6.7423(3)	13.2691(6)	90	1498.63(11)	2
5	400	*Imma*	16.7594(6)	6.7387(2)	13.2554(5)	90	1497.02(11)	2
6	375	*Imma*	16.7526(5)	6.7411(2)	13.2577(5)	90	1497.2(8)	2
7	350	*Imma*	16.7590(6)	6.7402(2)	13.2480(5)	90	1496.48(9)	2
8	325	*Imma*	16.7655(5)	6.7400(2)	13.2414(5)	90	1496.27(8)	2
9	300	*I2/a*	6.7061(8)	6.914(2)	19.3223(19)	95.68(1)	891.5(3)	4
10	275	*I2/a*	6.7262(16)	6.867(4)	19.348(4)	95.81(2)	889.0(5)	4
11	250	*I2/a*	6.726(4)	6.844(7)	19.329(9)	95.31(5)	885.9(11)	3
12	225	*I2/a*	6.7053(4)	6.8117(6)	19.3338(9)	95.990(5)	878.24(10)	3
13	200	*I2/a*	6.7043(4)	6.7840(6)	19.3325(10)	96.066(5)	874.36(10)	4
14	175	*I2/a*	6.7034(4)	6.7528(7)	19.3329(11)	96.162(5)	870.08(12)	3
15	150	*I2/a*	6.7042(5)	6.7308(7)	19.3312	96.223(5)	867.17(12)	3
16	125	*I2/a*	6.7065(4)	6.7094(7)	19.3264(9)	96.257(5)	864.44(11)	3
17	100	*I2/a*	6.7075(7)	6.6881(12)	19.3308(17)	96.330(8)	861.9(2)	2
18	473	*I2/a*	6.6987(14)	7.195(3)	19.275(3)	94.928(16)	925.6(5)	4

Table S2: Summary of the twin law and relative rotation angle between components, and number of peaks indexed to each component for the variable temperature data collection for int-**1**·0.928(8)py over the temperature range 100 - 473 K.

Sequence number (corresponding to Figure 3)	Twin law between components (direct space)	Relative twin rotation angle between components (°)	Peaks indexed to Component 1 (UB1) (%)	Peaks indexed to Component 2 (UB2) (%)	Peaks indexed to Component 3 (UB3) (%)	Peaks indexed to Component 4 (UB4) (%)	Unindexed Peaks (%)
1	[1.00 -0.00 -0.00]	Rot(UB1,UB2)=-179.9962	55.1	32.9	/	/	12
2	[0.01 0.86 0.51] (UB1:UB2)<br>[0.00 0.02 1.00] (UB1:UB3)<br>[-0.01 -0.03 1.00](UB1:UB4)<br>[1.00 -0.00 -0.00](UB2:UB3)<br>[1.00 0.00 0.00](UB2:UB4)<br>[-0.99 0.06 -0.09](UB3:UB4)	Rot(UB1,UB2)=179.1415<br>Rot(UB1,UB3)=-179.9505<br>Rot(UB1,UB4)=179.0354<br>Rot(UB2,UB3)=-104.4859<br>Rot(UB2,UB4)=-108.9498<br>Rot(UB3,UB4)=4.6165	24.1	13.8	14.4	11.3	36
3	[0.01 1.00 -0.01]	Rot(UB1,UB2)=-179.5923	74.2	15.4	/	/	10.4
4	[-0.00 0.71 0.71]	Rot(UB1,UB2)=179.8825	73.2	9.5	/	/	17.3
5	[1.00 0.00 -0.00]	Rot(UB1,UB2)=103.2319	74.7	10.8	/	/	14.43
6	[1.00 -0.00 0.00]	Rot(UB1,UB2)=103.2969	74.5	10	/	/	15.5
7	[1.00 -0.00 -0.00]	Rot(UB1,UB2)=103.4028	76.2	9.8	/	/	14
8	[0.01 0.01 1.00]	Rot(UB1,UB2)=179.5296	77	13.1	/	/	9.9
9	[0.00 1.00 -0.02] (UB1:UB2)<br>[1.00 -0.02 0.01] (UB1:UB3)<br>[0.28 0.04 0.96] (UB1:UB4)<br>[0.25 0.04 0.97](UB2:UB3)<br>[1.00 -0.01 -0.01](UB2:UB4)<br>[0.03 1.00 0.01](UB3:UB4)	Rot(UB1,UB2)=178.1282<br>Rot(UB1,UB3)=174.5934<br>Rot(UB1,UB4)=-179.5345<br>Rot(UB2,UB3)=-177.9563<br>Rot(UB2,UB4)=174.4101<br>Rot(UB3,UB4)=176.8992	39	19	10.8	7.5	24
10	[-0.08 1.00 -0.02](UB1:UB2)<br>[0.65 0.74 0.20](UB1:UB3)<br>[1.00 0.01 -0.00](UB1:UB4)<br>[-0.06 1.00 -0.03](UB2:UB3)<br>[0.16 0.08 0.98](UB2:UB4]<br>[1.00 0.00 0.02](UB3:UB4)	Rot(UB1,UB2)=-177.5475<br>Rot(UB1,UB3)=4.5094<br>Rot(UB1,UB4)=178.7147<br>Rot(UB2,UB3)=-179.606<br>Rot(UB2,UB4)=-174.8300<br>Rot(UB3,UB4)=176.3082	16.8	25.4	11.1	14.7	32
11	[1.00 -0.04 0.02](UB1:UB2)<br>[0.06 1.00 0.03](UB1:UB3)<br>[0.15 -0.11 0.98](UB2:UB3)	Rot(UB1,UB2)=-175.5353<br>Rot(UB1,UB3)=-179.7109<br>Rot(UB2,UB3)=-179.3107	31.3	19.9	10.8	/	38
12	[1.00 -0.08 0.01](UB1:UB2)<br>[0.98 -0.06 -0.21](UB1:UB3)<br>[1.00 -0.03 0.01](UB2:UB3)	Rot(UB1,UB2)=-174.4631<br>Rot(UB1,UB3)=10.8444<br>Rot(UB2,UB3)=176.3214	21.8	13.4	9	/	56

Table S2: continued

Sequence Number (corresponding to Figure 3)	Twin law between components (direct space)	Relative twin rotation angle between components (°)	Peaks indexed to Component 1 (UB1) (%)	Peaks indexed to Component 2 (UB2) (%)	Peaks indexed to Component 3 (UB3) (%)	Peaks indexed to Component 4 (UB4) (%)	Unindexed Peaks (%)
13	[0.78 -0.56 -0.26](UB1:UB2)<br>[0.98 0.04 -0.21](UB1:UB3)<br>[1.00 -0.08 0.01](UB1:UB4)<br>[0.61 0.79 -0.06](UB2:UB3)<br>[1.00 -0.03 -0.01](UB2:UB4)<br>[1.00 -0.03 0.01](UB3:UB4)	Rot(UB1,UB2)=8.6252<br>Rot(UB1,UB3)=10.6631<br>Rot(UB1,UB4)=-174.5239<br>Rot(UB2,UB3)=5.2523<br>Rot(UB2,UB4)=179.4310<br>Rot(UB3,UB4)=176.5343	20	10.2	9.8	10.2	50
14	[0.20 -0.13 0.97](UB1:UB2)<br>[1.00 -0.05 -0.00](UB1:UB3)<br>[-0.03 1.00 -0.01](UB2:UB3)	Rot(UB1,UB2)=171.1017<br>Rot(UB1,UB3)=-170.9861<br>Rot(UB2,UB3)=175.1952	21.5	13.7	7.2	/	65
15	[1.00 -0.05 0.02](UB1:UB2)<br>[0.98 0.04 -0.21](UB1:UB3)<br>[1.00 -0.00 0.02](UB2:UB3)	Rot(UB1,UB2)=-175.6988<br>Rot(UB1,UB3)=10.6128<br>Rot(UB2,UB3)=-175.3641	19.5	14.7	8.4	/	57
16	[0.74 0.67 0.01](UB1:UB2)<br>[0.17 -0.10 0.98](UB1:UB3)<br>[0.72 -0.70 0.03](UB2:UB3)	Rot(UB1,UB2)=-177.3259<br>Rot(UB1,UB3)=174.3684<br>Rot(UB2,UB3)=176.4404	16.4	5.9	11.1	/	67
17	[0.22 -0.17 0.96]	Rot(UB2,UB3)=176.4404	27.4	6.8	/	/	78
18	[0.09 0.15 0.98](UB1:UB2)<br>[0.14 0.11 0.98](UB1:UB3)<br>[-0.94 0.02 0.33](UB1:UB4)<br>[-0.76 -0.60 0.24](UB2:UB3)<br>[0.02 1.00 0.01](UB2:UB4)<br>[0.06 1.00 0.00](UB3:UB4)	Rot(UB1,UB2)=-172.9887<br>Rot(UB1,UB3)=-171.0664<br>Rot(UB1,UB4)=-169.6999<br>Rot(UB2,UB3)=3.5262<br>Rot(UB2,UB4)=88.8079<br>Rot(UB3,UB4)=90.7201	32.6	10.3	15.5	13.6	28

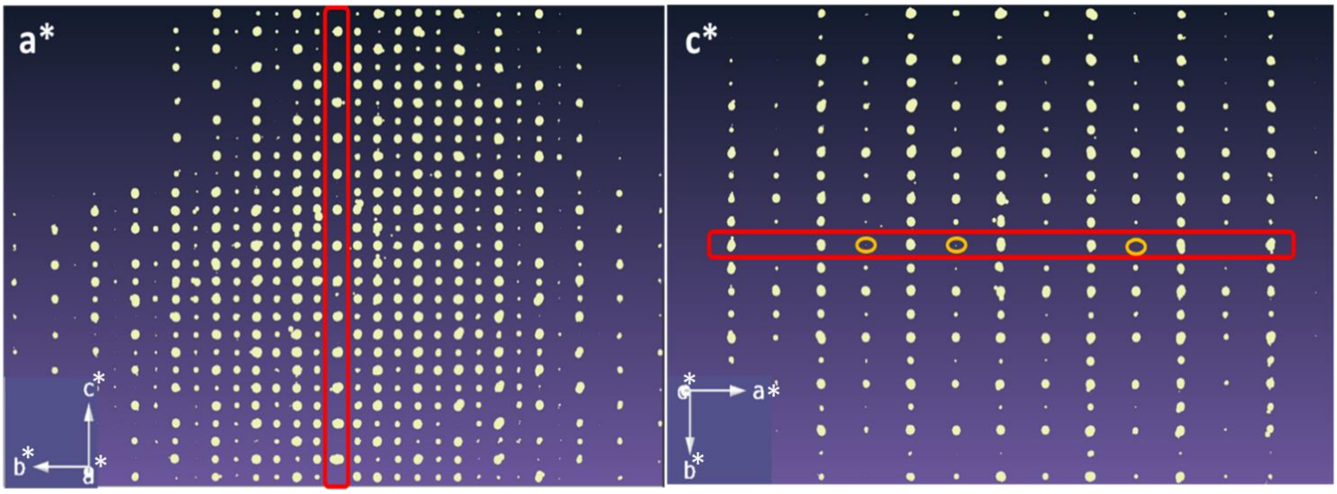

Figure S1: Ewald sphere projections along the reciprocal lattice vectors *a** (left) and *c** (right) for int-**1**·H_2O in the conventional cell setting *a* = 6.6833(4) Å, *b* = 14.8731(13) Å, *c* = 19.2890(13) Å, β = 96.226(7)° (*P*2_1*/c*). A *c*-glide perpendicular to *b** can be observed in the Ewald sphere projection along the a* axis (left), and a pseudo *a*-glide (also perpendicular to b*) is seen in the Ewald sphere projection along the c* axis (right) as highlighted by the red boxes. The weak peaks, as highlighted by the orange circles, break the *a*-glide symmetry. In all, this indicates that the correct space group choice is *P*2_1*/c* in the conventional cell setting.

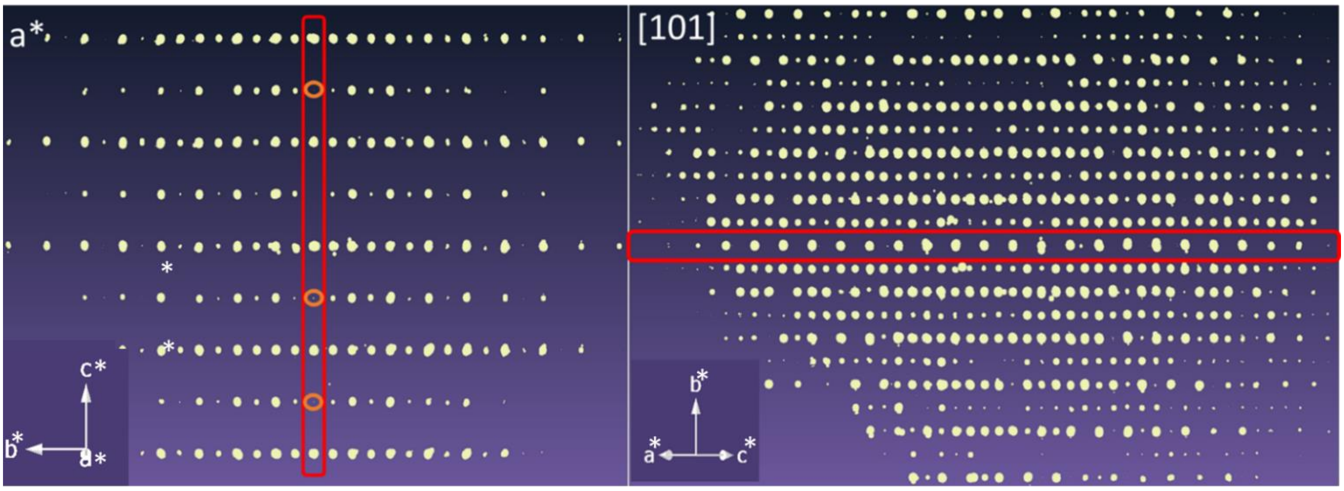

Figure S2: Ewald sphere projections along the reciprocal lattice vectors *a** (left) and [101] (right) for int-**1**·H_2O in an alternative monoclinic setting and the previously reported unconventional unit cell *a* = 19.2890(13) Å, *b* = 14.8731(13) Å, *c* = 6.6833(4) Å, β = 103.453(7)° (*P*2_1*/n*). A *n*-glide perpendicular to *b** can be observed in the Ewald sphere projection along the [101] direction (right), and a pseudo *c*-glide (also perpendicular to b*) is seen in the Ewald sphere projection along the a* axis (left) as highlighted by the red boxes. The weak peaks, as highlighted by the orange circles, break the *c*-glide symmetry. In all, this indicates that the correct space group choice is *P*2_1*/n* in this alternative monoclinic setting with an unconventional cell setting.

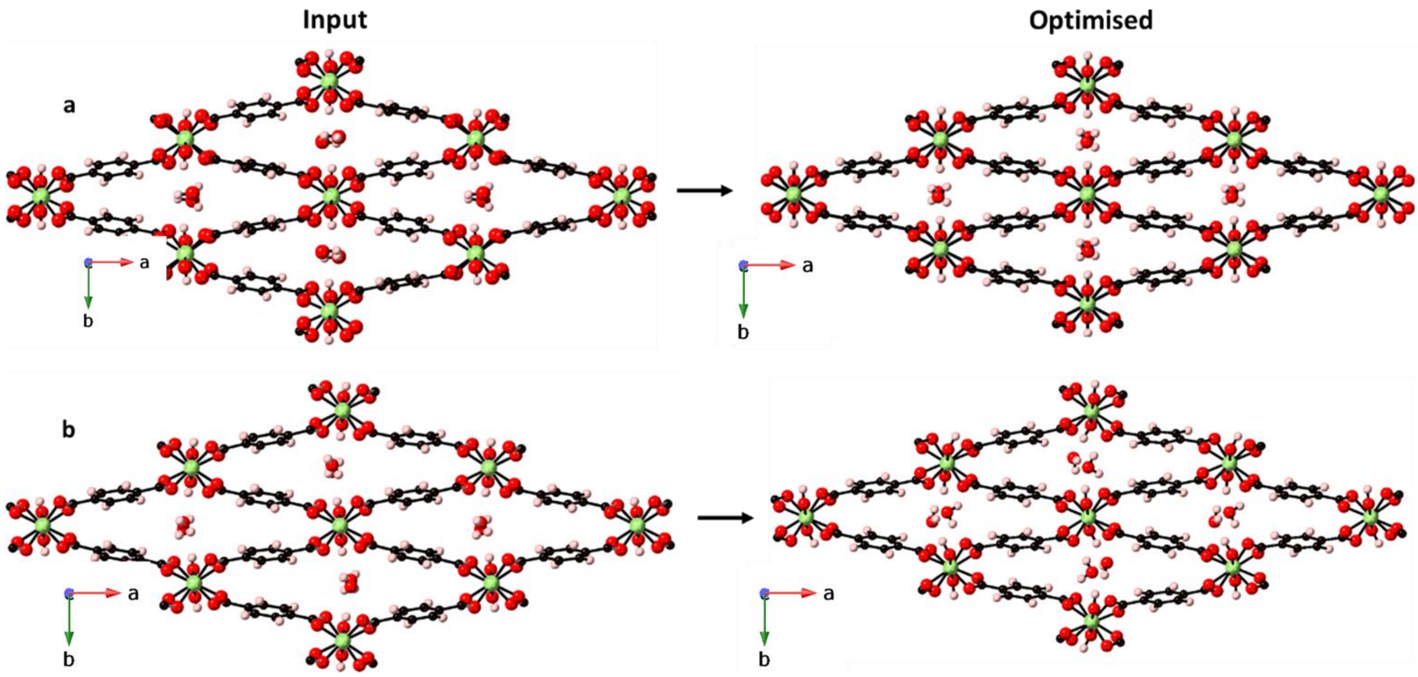

Figure S3: The pre- and post-optimised structures of (a) previously reported int-**1**·H_2O (*P2₁/c*)[Error! Bookmark not defined.] and (b) presently reported int-**1**·H_2O (*P2₁/n*). All structures are viewed along the [001] direction and represented in ball-and-stick mode. Atom key: green: Ga, red: O, black: C, pink: H.

Table S3: Unit cell parameters at specific temperatures for the variable temperature data collection for np-**1** over the temperature range 298 - 500 K.

Temperature/ K	*a*/ Å	*b*/ Å	*c*/ Å	*β*/ °	Volume/ Å^3
298	6.7086(12)	6.879(3)	19.311(2)	95.893(12)	886.5(4)
350	6.7134(10)	6.913(2)	19.338(2)	95.606(11)	893.2(3)
400	6.7145(9)	7.000(2)	19.3447(18)	95.428(10)	905.2(3
450	6.6954(7)	7.0865(16)	19.3077(15)	95.142(8)	912.4(2)
475	6.6950(8)	7.0865(16)	19.3077(15)	94.997(8)	918.8(3)
500	6.6993(6)	7.1934(13)	19.3048(11)	94.869(6)	926.95(19)

Table S4: Unit cell parameters for crystals of int-**1**·H_2O prior to flash heating.

Experiment Number	Temperature/ K	*a*/ Å	*b*/ Å	*c*/ Å	*β*/ °	Volume/ Å^3
1	100	6.632(9)	14.773(6)	19.225(6)	96.38(2)	1880(1)
2	100	Similar to above				
3	100	Similar to above				
4	100	Similar to above				
5	100	Similar to above				
6	100	6.6680(3)	14.7780(8)	19.2473(10)	96.326(4)	1885.07(17)

Table S5: Unit cell parameters for crystals of int-**1**·H_2O after flash heating to temperatures in the range of 375 - 500 K.

Experiment Number*	Temperature/ K	*a*/ Å	*b*/ Å	*c*/ Å	*β*/ °	Volume/ Å^3
1	375	6.7103(6)	6.9556(12)	19.3180(16)	95.570(7)	897.39(19)
2	400	6.7092(10)	7.002(3)	19.306(7)	95.55(2)	902.7(5)
3	425	6.7188(7)	7.0350(13)	19.3316(13)	95.334(8)	909.8(2)
4	450	6.7039(8)	7.0854(18)	19.3082(16)	95.171(8)	913.4(3)
5	475	6.720(2)	7.099(3)	19.309(5)	95.14(3)	917.4(6)
6	500	16.391(3)	6.7453(6)	13.794(3)	90	1525.2(4)

*Experiment number corresponds to values in Table S4.

Table S6: Unit cell parameters for a crystal of np-**1** at 350 K before flash heating and at 500 K after flash heating.

Temperature/ K	*a*/ Å	*b*/ Å	*c*/ Å	*β*/ °	Volume/ Å^3
350	6.7088(4)	6.9196(10)	19.3287(15)	95.568(7)	893.05(15)
500	6.7174(12)	7.178(2)	19.317(2)	94.857(13)	928.0(3)

Table S7: Summary of unit cell parameters and observed domains for the variable temperature data collection for int-**2**·0.84(3)py over the temperature range 100 - 500 K.

**Sequence Number (corresponding to Figure 6)**	**Temperature/ K**	**Space group**	***a*/ Å**	***b*/ Å**	***c*/ Å**	***β*/ °**	**Volume/ Å³**	**Number of Domains**
1	300	*I2/a*	6.7287(5)	11.0914(10)	18.0615(12)	91.893(6)	1347.21(18)	3
2 - major component	475	*Imma*	17.971(3)	6.6911(15)	11.151(3)	90	1340.8(6)	2
2* - minor component		*Imma*	16.728(18)	6.703(4)	13.08(2)	90	1467(3)	1
3	500	*Imma*	16.6641(15)	6.7191(7)	13.2001(17)	90	1478.0(3)	1
4	475	*Imma*	16.6601(13)	6.7203(6)	13.2040(15)	90	1478.3(2)	1
5	450	*Imma*	16.6655(13)	6.7175(5)	13.2065(14)	90	1478.5(2)	1
6	425	*Imma*	16.6659(13)	6.7185(6)	13.2105(15)	90	1479.2(2)	1
7	400	*Imma*	16.6739(12)	6.7163(5)	13.2064(14)	90	1478.9(2)	1
8	375	*Imma*	16.6784(12)	6.7162(5)	13.2022(13)	90	1478.9(2)	1
9	350	*Imma*	16.6833(11)	6.7181(5)	13.1983(12)	90	1479.3(2)	1
10	325	*Imma*	16.6954(11)	6.7183(5)	13.1906(13)	90	1479.5(2)	1
11	300	*Imma*	16.7023(9)	6.7201(4)	13.1821(11)	90	1479.57(17)	1
12	275	*Imma*	16.7093(10)	6.7203(4)	13.1767(11)	90	1479.63(18)	1
13	250		6.698(3)	7.008(12)	19.237(8)	95.56(5)	898.7(16)	N/A
14*	225		6.698(7)	6.94(2)	19.271(18)	95.58(10)	891(3)	N/A
15*	200		6.701(10)	6.89(2)	19.26(2)	95.78(11)	885(3)	N/A
16*	175		6.679(9)	6.878(18)	19.237(16)	95.73(14)	879(3)	N/A
17*	150		6.682(8)	6.819(19)	19.278(16)	95.80(10)	874(3)	N/A
18*	125		6.736(13)	6.85(3)	19.25(2)	95.5(2)	884(4)	N/A
19	100	*Imma*	16.293(2)	6.7216(7)	13.701(2)	90	1500.5(3)	3
20	125	*Imma*	16.325(2)	6.7146(8)	13.673(2)	90	1498.9(4)	3
21	150	*Imma*	16.511(2)	6.7161(7)	13.427(2)	90	1488.9(3)	4
22*	175	*I2/a*	6.70(3)	8.7770(13)	18.990(13)	94.1(3)	1114(5)	N/A

*An insufficient number of reflections were observed to determine the crystal structure and the number of domains present so only the unit cell was determined.

Table S8: Summary of the twin law and relative rotation angle between components, and number of peaks indexed to each component for the variable temperature data collection for int-**2**·0.84(3)py over the temperature range 100 - 500 K.

Sequence number (corresponding to Figure 6)	Twin law between components (direct space)	Relative twin rotation angle between components (°)	Peaks indexed to Component 1 (UB1) (%)	Peaks indexed to Component 2 (UB2) (%)	Peaks indexed to Component 3 (UB3) (%)	Peaks indexed to Component 4 (UB4) (%)	Unindexed Peaks (%)
1	[0.01 1.00 0.03](UB1:UB2)<br>[0.01 1.00 0.04](UB1:UB3)<br>[-1.00 -0.09 -0.04](UB2:UB3)	Rot(UB1,UB2)=179.8431<br>Rot(UB1,UB3)=179.4360<br>Rot(UB2,UB3)=2.7274	38.5	28.7	19.9	/	26.3
2	[1.00 0.01 0.00] (UB1:UB2)<br>[-0.02 -0.03 1.00](UB1:UB3)<br>[-0.01 0.56 -0.83](UB2:UB3)	Rot(UB1,UB2)=-92.9177<br>Rot(UB1,UB3)=-179.5061<br>Rot(UB2,UB3)=179.8164	47.7	28.3	11.2	/	13
3	N/A	N/A	92.5	/	/	/	7.5
4	N/A	N/A	92.48	/	/	/	8
5	N/A	N/A	93.47	/	/	/	6.53
6	N/A	N/A	92.9	/	/	/	7.1
7	N/A	N/A	93.11	/	/	/	6.89
8	N/A	N/A	92.99	/	/	/	7.01
9	N/A	N/A	92.42	/	/	/	7.58
10	N/A	N/A	94.06	/	/	/	5.94
11	N/A	N/A	97.97	/	/	/	2.03
12	N/A	N/A	98.19	/	/	/	1.81
13	N/A	N/A	73	/	/	/	27
14*	N/A	N/A	N/A	N/A	N/A	N/A	N/A
15*	N/A	N/A	N/A	N/A	N/A	N/A	N/A
16*	N/A	N/A	N/A	N/A	N/A	N/A	N/A
17*	N/A	N/A	N/A	N/A	N/A	N/A	N/A
18*	N/A	N/A	N/A	N/A	N/A	N/A	N/A
19	[1.00 0.01 0.03](UB1:UB2)<br>[0.96 0.26 -0.11](UB1:UB3)<br>[0.99 0.06 0.11](UB2:UB3)	Rot(UB1,UB2)=-155.9430<br>Rot(UB1,UB3)=38.7652<br>Rot(UB2,UB3)=-171.9245	40.8	22.1	15.2	/	24.1
20	[-0.05 0.97 0.24](UB1:UB2)<br>[0.14 0.94 0.32](UB1:UB3)<br>[-0.56 0.67 0.49](UB2:UB3)	Rot(UB1,UB2)=173.5687<br>Rot(UB1,UB3)=-159.4437<br>Rot(UB2,UB3)=29.6894	39.3	19.5	14	/	27.3
21	[-0.12 -0.17 0.98](UB1:UB2)<br>[0.96 0.25 -0.11](UB1:UB3)<br>[0.37 -0.02 0.93](UB1:UB4)<br>[-0.41 0.06 0.91](UB2:UB3)<br>[0.84 -0.50 -0.19](UB2:UB4)<br>[0.01 0.21 0.98](UB3:UB4)	Rot(UB1,UB2)=-177.8761<br>Rot(UB1,UB3)=39.4244<br>Rot(UB1,UB4)=178.2799<br>Rot(UB2,UB3)=165.2354<br>Rot(UB2,UB4)=35.4260<br>Rot(UB3,UB4)=179.4695	38.8	20.2	15.1	9.9	21.7
22*	N/A	N/A	N/A	N/A	N/A	N/A	N/A

*An insufficient number of reflections were observed to determine the crystal structure and the number of domains present so only the unit cell was determined.

Table S9: Unit cell parameters at specific temperatures for the variable temperature data collection for np-**2** over the temperature range 100 - 500 K.

Temperature/ K	*a*/ Å	*b*/ Å	*c*/ Å	*β*/ °	Volume/ $Å^3$
100	6.6662(3)	14.7953(10)	19.2119(8)	96.366(4)	1883.16(17)
350	6.709(3)	6.935(3)	19.342(10)	95.53(5)	895.7(7)
375	6.733(6)	6.990(5)	19.281(19)	96.11(11)	902(1)
400	6.692(8)	7.019(5)	19.32(4)	95.35(12)	903(2)
425	6.7032(8)	7.057(2)	19.314(2)	95.283(12)	909.8(3)
450	6.706(5)	7.120(4)	19.389(18)	95.28(6)	922(1)
475	6.724(3)	7.160(2)	19.303(10)	95.06(4)	925.8(7)
500	6.702(7)	7.185(5)	19.41(2)	94.94(9)	931(1)

Table S10: Unit cell parameters for crystals of int-**2**·H_2O prior to flash heating.

Experiment Number	Temperature/ K	*a*/ Å	*b*/ Å	*c*/ Å	*β*/ °	Volume/ $Å^3$
1	150	6.67338(14)	14.8533(5)	19.2365(5)	96.257(2)	1895.39(9)
2	150	6.67644(13)	14.8632(5)	19.2238(4)	96.221(2)	1896.41(8)
3	150	6.6682(6)	14.8475(11)	19.2305(14)	96.229(6)	1892.7(3)
4	150	6.6697(4)	14.8565(9)	19.2581(13)	96.288(5)	1896.8(2)
5	150	6.6736(2)	14.8625(6)	19.2504(7)	96.238(3)	1898.06(13)
6	100	6.6664(2)	14.8002(8)	19.2350(8)	96.287(3)	1886.39(14)

Table S11: Unit cell parameters for crystals of int-**2**·H_2O after flash heating to temperatures in the range of 375-500 K.

Experiment Number*	Temperature/ K	*a*/ Å	*b*/ Å	*c*/ Å	*β*/ °	Volume/ $Å^3$
1	375	6.7129(4)	6.9937(9)	19.3215(12)	95.474(6)	902.96(14)*
2	400	6.7058(5)	7.0231(9)	19.3172(15)	95.392(7)	905.72(15)
3	425	6.7136(5)	7.0580(14)	19.3234(15)	95.321(7)	911.7(2)
4	450	6.7167(4)	7.1153(13)	19.3334(10)	95.187(6)	920.18(18)
5	475	6.7145(3)	7.1468(9)	19.3117(11)	95.073(5)	923.07(13)
6	500	16.669(2)	6.7505(11)	13.360(4)	90	1503.3(5)**

*Experiment number corresponds to values in Table SI0.

Table S12: Unit cell parameters for a crystal of np-**2** at 350 K before flash heating and at 500 K after flash heating.

Temperature/ K	*a*/ Å	*b*/ Å	*c*/ Å	*β*/ °	Volume/ $Å^3$
350	6.7062(5)	6.9462(11)	19.3256(13)	95.620(7)	895.91(17)
500	6.7146(9)	7.228(3)	19.309(2)	94.829(9)	933.9(4)

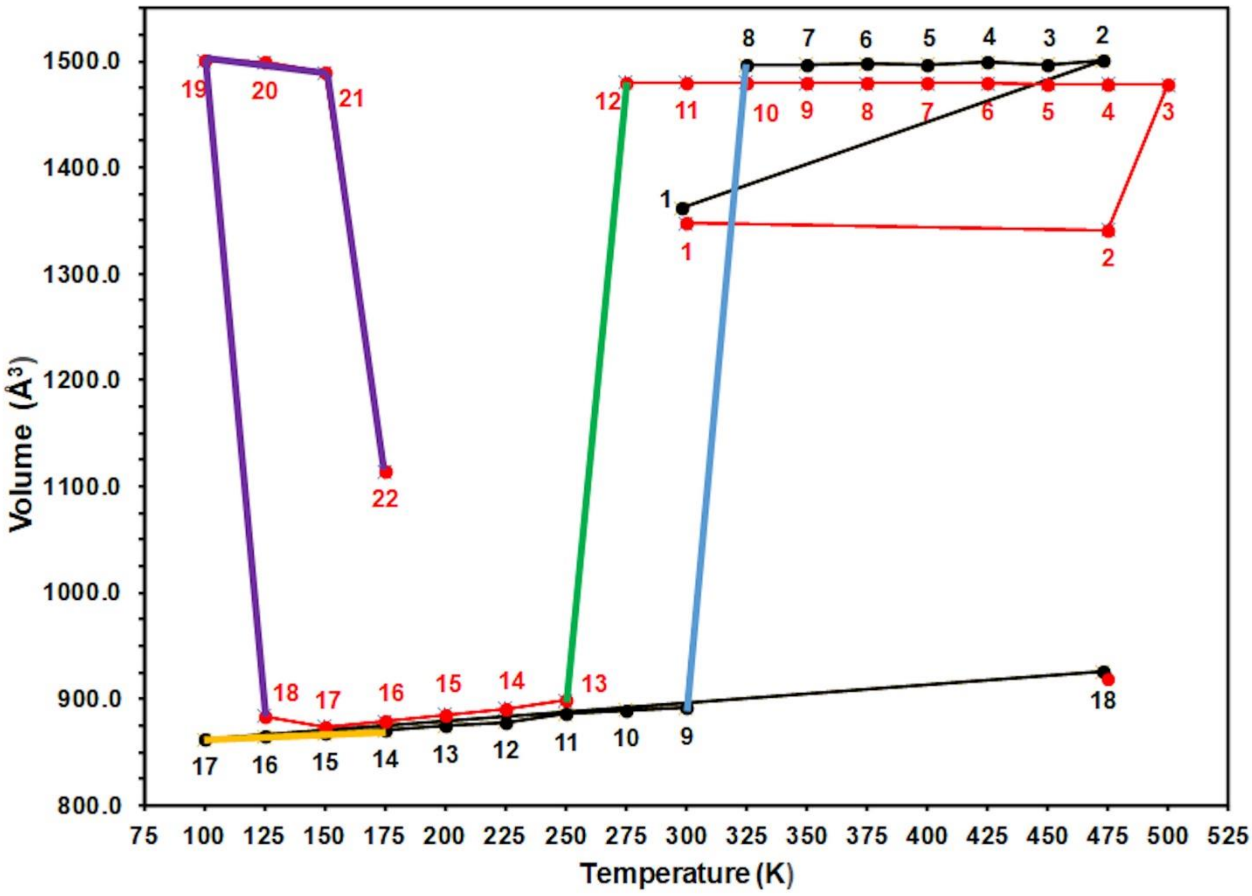

Figure S4: Comparison of thermoresponsive behaviour of int-**1**·0.928(8)py (black lines and numbers) and int-**2-**·0.84(3)py (red lines and numbers) over the temperature range of 100 – 500 K. The difference in the lp to np phase transition temperature is highlighted in blue and green for **1** and **2** respectively and the difference in low temperature behaviour is highlighted in orange and purple for **1** and **2** respectively. From this plot it can be seen that int-**2**·0.84(3)py undergoes the lp to np transition between 275 - 250 K which is 50 K lower than the equivalent phase transition for int-**1**·0.928(8)py, additionally int-**2**·0.84(3)py exhibits a low temperature np to lp reopening between 125 - 100 K which is not observed for int-**1**·0.928(8)py.

Table S13: List of CCDC deposition number and corresponding structure.

**Deposition Number**	**Structure**
2223704	GaOH_py_100_percent
2223705	GaOH_py_1_300
2223706	GaOH_py_2_473
2223707	GaOH_py_3_450
2223729	GaOH_py_4_425
2223730	GaOH_py_5_400
2223731	GaOH_py_6_375
2223732	GaOH_py_7_350
2223733	GaOH_py_8_325
2223710	GaOH_py_9_300
2223711	GaOH_py_10_275
2223716	GaOH_py_11_250
2223721	GaOH_py_12_225
2223722	GaOH_py_13_200
2223723	GaOH_py_14_175
2223724	GaOH_py_15_150
2223725	GaOH_py_16_125
2223726	GaOH_py_17_100
2223727	GaOH_py_18_473
2223728	GaOH_H2O
2223735	GaOHF_py_01
2223736	GaOHF_py_02
2223737	GaOHF_py_03
2223738	GaOHF_py_04
2223739	GaOHF_py_05
2223740	GaOHF_py_06
2223741	GaOHF_py_07
2223742	GaOHF_py_08
2223743	GaOHF_py_09
2223744	GaOHF_py_10
2223745	GaOHF_py_11
2223746	GaOHF_py_12
2223747	GaOHF_py_13
2223748	GaOHF_py_19
2223749	GaOHF_py_20
2223750	GaOHF_py_21
2223751	GaOHF_H2O
2223752	GaOHF_dehydrated_flash1
2223753	GaOHF_dehydrated_flash2
